# STOP-Bang–Defined Obstructive Sleep Apnea Risk and Postoperative Nausea and Vomiting in Laparoscopic Surgery Patients: A Prospective Cohort Study

**DOI:** 10.7759/cureus.100038

**Published:** 2025-12-24

**Authors:** Nauman Haider, Atta Okasha, Ayesha Mansoor, Kayode A Babasanya, Riaz Ahmed, Faryal Ali, Umair Majeed, Muhammad Faizan Khan Khalil, Asaad Hussein, Shaumile H Khan, Hiba Malik

**Affiliations:** 1 Urology, Jinnah Hospital, Lahore, PAK; 2 Internal Medicine, Arab Academy for Management, Banking and Financial Sciences (AAMBFS), Cairo, EGY; 3 Medicine, Faisalabad Medical University, Faisalabad, PAK; 4 Emergency Medicine, Medway Maritime Hospital, Gillingham, GBR; 5 Surgery, Liaquat University of Medical and Health Sciences, Jamshoro, PAK; 6 General Surgery, Pakistan Institute of Medical Sciences, Islamabad, PAK; 7 Medicine, Holy Family Red Crescent Medical College Hospital, Dhaka, BGD; 8 Surgery, Combined Military Hospital, Kohat, PAK; 9 General Practice, Ankara Yıldırım Beyazıt University, Ankara, TUR; 10 Medicine, Fatima Memorial Hospital College of Medicine and Dentistry, Lahore, PAK; 11 Internal Medicine, Central Park Medical College, Lahore, PAK

**Keywords:** apfel score, laparoscopic surgery, obstructive sleep apnea, perioperative risk, postoperative nausea and vomiting, stop-bang

## Abstract

Background: Obstructive sleep apnea (OSA) is frequently underdiagnosed and may influence perioperative outcomes. Postoperative nausea and vomiting (PONV) are common adverse effects following laparoscopic surgery. The purpose of this study was to determine the relationship between PONV and the risk or predisposition of OSA based upon STOP-Bang criteria in laparoscopic surgery patients.

Methods: This is a prospective cohort study conducted in government and private hospitals in Lahore from January to July 2025. The sample consisted of 200 patients who underwent elective laparoscopic surgery under general anesthesia, recruited via convenience sampling. The OSA risk was categorised using the STOP-Bang questionnaire, whereas PONV was measured by the Apfel simplified risk score and the visual analogue scale (VAS). Statistical analyses were performed using SPSS v.26, including descriptive statistics, Spearman correlations, Mann-Whitney U tests, Kruskal-Wallis tests, chi-square tests, and multiple linear regression analyses, while controlling for age, gender, medication use, surgical history, and anesthesia history.

Results: Among the 200 participants, 152 (76%) were female, and 48 (24%) were male. The most common age ranges were 56-60 years (73 participants, 36%) and 46-55 years (54 participants, 27%). The relationships among the Apfel, VAS, and STOP-Bang scores showed moderately positive correlations (r = 0.23, p < 0.01; r = 0.19, p < 0.01). Regression analysis showed that STOP-Bang scores (beta = 0.152, p = 0.003), nausea severity (beta = 0.176, p = 0.003), female gender (beta = 0.125, p = 0.008), and medication use (beta = 0.142, p = 0.003) were associated with higher Apfel scores. Younger age was associated with higher PONV scores (χ² = 21.77, p = 0.001). Female patients scored significantly higher on the STOP-Bang, Apfel, and VAS than males (p < 0.05).

Conclusion: STOP-Bang-defined OSA risk has a significant association with PONV in laparoscopic surgery patients. Female gender and younger age increased susceptibility to PONV, whereas OSA risk was augmented in older people. These results illustrate the clinical utility of integrating STOP-Bang screening into preoperative evaluation to optimise perioperative care and individualise preventive measures against PONV. These findings support integrating STOP-Bang screening into preoperative risk assessment to improve postoperative outcomes.

## Introduction

Obstructive sleep apnea (OSA) is a chronic disorder caused by repeated collapse of the airway during sleep, leading to apneas, hypopneas, and systemic effects such as neurocognitive impairment, hypertension, cardiovascular disease, and metabolic dysfunction. It affects approximately 3-7% of adults, with 2-4% of middle-aged adults being affected, but the majority of cases remain undiagnosed, underscoring the need for improved screening and awareness [1-3].

OSA is closely associated with obesity and contributes to neurocognitive impairment, hypertension, and heart failure. Diagnosis relies on screening tools and polysomnography, with continuous positive airway pressure (CPAP) being the most effective therapy despite compliance challenges [4,5]. Among surgical patients, moderate-to-severe OSA is present in 31% of cases, yet it remains undetected among surgeons (92%) and anesthetists (60%), highlighting the importance of improved preoperative screening [6]. The STOP-Bang questionnaire is an effective screening measure for obstructive sleep apnea (OSA) in various populations. It has been demonstrated that this tool is potentially effective in a sleep clinic setting for diagnosing and prioritising patients at high risk of moderate-to-severe OSA [7].

Postoperative nausea and vomiting (PONV) are one of the primary issues in anaesthesia and have a multifactorial cause, which includes anaesthetic drugs (nitrous oxide, opioids, and older inhalation agents), and patient- and surgery-associated factors [8,9]. PONV develops in approximately 52% of patients during the first 24 hours after surgery, and vomiting occurs in approximately 25%. The incidence rates are greater following general anaesthesia (52%) than regional anaesthesia (38%). Other predictive factors include female gender, prior PONV, motion sickness, nonsmoking status, and longer surgery duration [10].

The latest PONV guidelines, informed by an international expert panel guided by the Society for Ambulatory Anaesthesia, provide evidence-based methods for identifying high-risk patients, minimising base risks, and optimising both pharmacological and non-pharmacological prophylaxis and treatment [11].

Since the risk of OSA is high in surgical patients and PONV imposes a significant clinical burden, insights into this relationship may be highly relevant for stratifying postoperative risk, planning perioperative care, and maximising perioperative outcomes. Thus, this prospective study seeks to determine the association between OSA risk, as defined by STOP-Bang, and the occurrence of postoperative nausea and vomiting in the population of patients undergoing laparoscopic surgery. We hypothesized that higher STOP-Bang scores would independently predict an increased risk of PONV in this patient population.

Rationale

Obstructive sleep apnea (OSA) is well established in surgical patients, but most research has focused on perioperative respiratory and cardiovascular outcomes, with little attention to postoperative nausea and vomiting (PONV). The STOP-Bang questionnaire is a validated tool for identifying patients at risk of OSA; however, its utility in predicting PONV has not been explored. PONV contributes to patient dissatisfaction, delayed discharge, and increased healthcare costs, making it clinically essential to determine whether OSA risk is an independent factor, particularly in laparoscopic surgery, where PONV rates are high. With obesity and other OSA risk factors rising in the Pakistani population, the burden of untreated OSA is likely significant. Given the limited local data, this study aims to evaluate the association between OSA risk and PONV severity, asking whether OSA risk is independently associated with PONV severity after adjusting for demographic and clinical factors. The findings could inform perioperative screening, risk stratification, and management strategies in local practice.

Objectives

Primary Objectives

To determine the association between obstructive sleep apnea (OSA), as defined by the STOP-Bang score, and the occurrence of postoperative nausea and vomiting (PONV) in patients undergoing laparoscopic surgery.

Secondary Objectives

To explore whether patient-related factors (e.g., age, gender) and OSA risk scores can predict the likelihood or severity of PONV, thereby informing perioperative risk stratification.

## Materials and methods

Study design

The study examined the relationship between obstructive sleep apnea (OSA) risk identified using STOP-Bang and postoperative nausea and vomiting (PONV) in patients with laparoscopic surgery using a prospective cohort study design. The patients were selected from the surgery wards and preoperative outpatient clinics of public and private hospitals in Lahore, resulting in a varied selection of socioeconomic and cultural backgrounds.

The OSA risk was assessed using a structured questionnaire, which included the STOP-Bang screening tool, administered before surgery. The incidence and severity of nausea and vomiting during the hospital stay were measured using validated assessment scales postoperatively. Patients were approached during the routine preoperative assessment by trained research assistants who explained the study's aims and methodology, and obtained written or verbal informed consent. The patients were supported by a research team member or answered the questionnaire themselves when possible, depending on their literacy level. This methodology provided reliable and accurate data collection, while also ensuring culturally respectful and sensitive communication in the local care setting.

Sampling strategy and population size

The sample size was calculated using the World Health Organisation (WHO) formula to estimate a population proportion with a 95% confidence interval and a 5% margin of error, assuming an unknown population proportion, a specified margin of error, and a 0.5 proportion of the population [12]. Based on these parameters, the minimum population size was estimated to be approximately 384 participants.

Due to the limited data-collection period (April-July 2025), variable daily patient flow across participating surgical units, and the exclusion of incomplete or inaccurately completed questionnaires, the final analyzable sample consisted of 200 participants. The reduced sample size is acknowledged as a limitation; however, despite this limitation, we were able to detect statistically significant associations. The findings should be interpreted with caution, given the smaller sample and potential limitations in generalizability.

Inclusion criteria

The study was done on patients aged 18 years and above who needed elective laparoscopic surgery using general anaesthesia and were willing to sign the informed consent. Patients had to be able to read and complete the preoperative questionnaire independently or with assistance from a research team member.

Exclusion criteria

Individuals who had a previous history of chronic nausea or vomiting, gastrointestinal illnesses that involve nausea and vomiting, a history of undergoing bariatric or major upper abdominal surgery, known neurologic/psychological disorders that may hinder the recording of symptoms, or those who were preoperatively on antiemetic medication were excluded. Patients who refused to take Part were also excluded.

Data collection tools

A structured questionnaire was designed to collect relevant and comprehensive data in this study, comprising three main parts: demographic data, OSA risk assessment, and postoperative nausea and vomiting (PONV) assessment. The main aim of the questionnaire was to determine the correlation between OSA risk as defined by STOP-Bang and incidence and severity of PONV amongst patients who undergo laparoscopic surgery. All of the standardised instruments were in their native English language, and hence participants were able to answer accurately because no cultural or linguistic adaptations were necessary.

The STOP-Bang questionnaire

The STOP-Bang questionnaire is a validated screening tool developed by Chung et al. in 2008 to assess obstructive sleep apnea (OSA) in preoperative and clinical settings. It includes eight questions: Snoring, Tiredness, observed apnea, high blood Pressure, Body mass index, Age, Neck circumference, and Gender, all coded 0 (not present) or 1 (present) and range in value between 0 and 8. The participants are categorised according to their risk of OSA as low (0-2), intermediate (3-4), or high (5-8). The questionnaire exhibits good internal consistency, with an estimated Cronbach's alpha ranging from 0.70 to 0.75. This research categorised patients by risk of OSA using the STOP-Bang before undergoing laparoscopic surgery and examined the relationship between preoperative OSA-risk stratification and postoperative PONV. It was a short-form, easily administered, and had a proven predictive value, making it suitable for application in the preoperative clinical arena [13]. Permission to use the STOP-Bang Questionnaire, originally developed by Dr. Frances Chung (University of Toronto), was obtained via email correspondence with the developer (see Figure 1 in the Appendix).

Apfel score

It was developed by Dr. Christian Apfel and others in 1999. The Apfel Score is a validated, yet simple, risk assessment of postoperative nausea and vomiting (PONV) after surgical procedures in surgical patients. It includes four items: female sex, history of motion sickness or prior PONV, non-smoking status, and postoperative opioid use. All of them are given 1 point, making the maximum score four and indicating that the level of PONV risk increases with the score. The Apfel Score has shown good internal consistency, with Cronbach's alpha values reported to be approximately 0.65-0.75. The Apfel Score in this study was used to classify patients into different risk groups for developing PONV. However, because patients with a prior history of PONV were excluded from the study, the overall risk predicted by the scale may have been slightly underestimated. Thus, relationships between preoperative OSA risk and the occurrence of nausea and vomiting during and after surgery were analysed in patients undergoing laparoscopic surgery [14].

Visual analogue scale (VAS)

Subjective symptoms were assessed using a Visual Analogue Scale (VAS), as described by Aitken (1969). The VAS commonly comprises a single 10-cm horizontal scale, anchored by 'no nausea/vomiting' at zero and the worst possible nausea/vomiting at 10, as used in the assessment of postoperative nausea and vomiting (PONV). Patients report their severity on a line, which is recorded in centimetres to assign a score between 0 and 10. The VAS is commonly known to be easy to use and sensitive to changes in symptom severity. Internal consistency is typically lower for single-item VAS reports; however, reliability studies have shown high test-retest correlation coefficients (r > 0.80), indicating strong reproducibility. The VAS was used to clinically measure the severity of nausea and vomiting in the postoperative period, enabling a precise comparison of the experience strength across patients with varying OSA risk levels [15].

Procedure

The study participants were identified in the preoperative clinics and surgical units of tertiary-level hospitals, as well as in government and privately owned hospitals in Lahore. The data collection took place over four months, from April to July 2025. Eligible patients who were undergoing elective laparoscopic surgery were recruited through regular preoperative visits during the assessment period and were informed adequately about the aim, rationale, and design of the study in their native language to minimise the risk of miscommunication. All data collection was conducted after written or verbal informed consent was obtained. Particular consideration was given to preserving privacy and confidentiality; no personal identifiers were stored, and the answers were processed anonymously. This method enabled a thorough and ethically conscious data-gathering process and addressed the needs of a multicultural patient group.

Analytical approach

Data analysis was done in IBM SPSS Statistics 26 (IBM Corp.). Descriptive statistics, including frequencies and percentages, were used to describe the demographic and clinical characteristics of the 200 participants. Normality of the key study variables, including the STOP-Bang questionnaire (SBQ), APFEL Simplified Risk Score, and Visual Analogue Scale (VAS) scores, was determined through detrended Normal-Q-Q plots. The non-parametric tests were used since the data were not normally distributed. To determine the correlation between SBQ, APFEL, and VAS scores, Spearman correlation analysis was used. Gender differences in these scores were compared using the Mann-Whitney U test. The Kruskal-Wallis test was used to compare scores across age groups and medication-use types. Multiple linear regressions were also conducted to determine the predictors of postoperative outcomes, which included SBQ and VAS scores, as well as demographic and clinical predictors: age, sex, marital status, work status, medical history, medications, OSA Diagnosis, PONV history, surgical and anaesthesia history, and antiemetic drug use. The relationship between age groups and categorical variables, specifically diagnosed/evaluated OSA and postoperative nausea/vomiting (PONV), was tested using the chi-square test. All statistical tests were two-sided, and the significance level was set at p < 0.05.

Ethical protocols

The research was conducted in accordance with the ethical principles governing the study of human subjects. The Central Park Medical College Institutional Review Board CPMC/IRB/2025-909 reviewed and approved the research protocol. The study adhered to core ethical principles that uphold the values of respecting persons, beneficence, and confidentiality. It was made clear to the participants that they could opt out of the study at any time without affecting their clinical treatment. Written informed consent was obtained from literate participants, and verbal consent was obtained from illiterate participants, as approved by the IRB. The data collection did not gather any personally identifiable information; all data was anonymised at the point of entry, processing, and analysis to maintain confidentiality. To maintain the integrity and reliability of the results, any incomplete replies to obligatory items were excluded from the final analysis. The study ensured the protection, dignity, and privacy of all the participants by incorporating these ethical considerations within the research process.

## Results

Table 1 presents the demographic and clinical characteristics of the participants (N = 200). Most were aged 46-60 years (n = 127, 63%), and the majority were female (n = 152, 76%). More than half were married (n = 110, 55%) and employed (n = 90, 45%). Common medical conditions included obstructive sleep apnea (n = 57, 29%), cardiovascular diseases (n = 47, 24%), and asthma (n = 39, 19%). Regarding medication use, blood pressure medications (n = 78, 39%) and diabetes medications (n = 54, 27%) were most frequently reported. A total of 30 participants (15%) had been diagnosed or evaluated for OSA. Postoperative nausea or vomiting (PONV) was frequently experienced by 90 participants (45%), while 102 (51%) reported previous anesthesia-related complications. Additionally, 139 participants (69%) indicated that they were taking anti-nausea medications for their current surgery.

**Table 1:** Demographic characteristics of participants (N = 200) Note. F = frequency, %=percentage; Values are presented as N (%), N = 200; No statistical comparisons were performed for demographic variables in this table

Variable	f (N)	%
Age		
18–25 years	10	5
26–35 years	5	3
36–45 years	7	4
46–55 years	54	27
56–60 years	73	36
61+ years	51	25
Gender		
Male	48	24
Female	152	76
Marital status		
Single	9	5
Married	110	55
Divorced	24	12
Widowed	57	28
Employment status		
Student	20	10
Employed	90	45
Unemployed	47	24
Retired	43	22
Do you have a history of any of the following medical conditions?		
Hypertension	6	3
Diabetes	22	11
Asthma	39	19
Cardiovascular diseases	47	24
Obstructive sleep apnea (OSA)	57	29
Chronic pain	25	12
None of the above	4	2
Medication use		
No regular medication	10	5
Opioids (e.g., morphine, oxycodone, etc.)	22	11
Antidepressants or anti-anxiety medication	34	17
Blood pressure medications	78	39
Insulin or other diabetes medications	54	27
Other	2	1
Have you ever been diagnosed with sleep apnea or evaluated for OSA?		
No	170	85
Yes	30	15
Do you have any history of postoperative nausea or vomiting (PONV)?		
No	5	3
Yes, frequent	90	45
Yes, sometimes	66	33
Never experienced surgery before	39	19
Surgical history		
None	12	6
Previous laparoscopic surgery	48	24
previous open surgery	105	52
Other	35	18
Anesthesia history		
No history of general anaesthesia	32	16
History of general anaesthesia without complications	60	30
History of complications under anaesthesia (e.g., nausea, vomiting)	102	51
not sure	6	3
Do you usually experience nausea after surgery?		
I usually experience nausea after surgery	77	39
I sometimes experience nausea after surgery	103	51
I never experience nausea after surgery	20	10
Are you taking any anti-nausea medications for this surgery?		
No	61	31
Yes	139	69

Table 2 shows the Spearman’s correlation coefficients among the SBQ, Apfel Simplified Risk Score, and VAS scores for the study sample (N = 200). A significant positive correlation was observed between SBQ and Apfel scores (r = 0.152, p = 0.003), as well as between SBQ and VAS scores (r = 0.18, p = 0.003). Similarly, the Apfel score was positively correlated with the VAS score (r = 0.176, p = 0.003). These findings indicate that higher OSA risk, as measured by the SBQ, is associated with increased postoperative nausea risk and greater symptom severity.

**Table 2:** Spearman's correlations among SBQ, Apfel Simplified Risk Score, and VAS scores (N = 200) Note. Values represent Spearman's correlation coefficients (r) between continuous variables; p < 0.01 (2-tailed) was considered statistically significant and is denoted with double asterisks (**).

Variables	STOP-Bang Questionnaire (SBQ)	Apfel Simplified Risk Score	Visual Analogue Scale (VAS)
STOP-Bang Questionnaire (SBQ)	-	r=0.152, p=0.003	r=0.18, p=0.003
Apfel Simplified Risk Score	-	-	r=0.176, p=0.003
Visual Analogue Scale (VAS)	-	-	-

Table 3 shows that females scored significantly higher on all three scales compared to males. In the case of the SBQ (U = 2788.000, Z = -2.412, p = 0.016), the female population (Mean Rank = 108.97) obtained higher scores than males (Mean Rank = 82.40). Likewise, the Apfel Simplified Risk Score was severer in females (Mean Rank = 108.38) than in males (Mean Rank = 84.10; U = 2870.000, Z = -2.218, p = 0.027). A significant gender difference also emerged when using Visual Analogue Scale (VAS) scores, with females (Mean Rank = 107.56) rating higher than males (Mean Rank = 86.50; U = 2985.000, Z = -2.001, p = 0.045). These findings show that the women members were more prone to report increased risk and burden of symptoms in all scales. These results indicate a statistically significant gender difference in STOP-Bang, Apfel, and VAS scores.

**Table 3:** Mann–Whitney U test comparing gender differences on the STOP-Bang Questionnaire, Apfel Simplified Risk Score, and Visual Analogue Scale (N = 200) Note. N = 200 (Males = 48, 24%; Females = 152, 76%); Mann–Whitney U test was used for all comparisons; p values marked with * indicate statistical significance at p <0.05.

Variable	Gender	N	Mean Rank	Sum of Ranks
STOP-Bang Questionnaire (SBQ)	Male	48	82.40	3955.00
-	Female	152	108.97	16,145.00
-	Total	200	-	-
Apfel Simplified Risk Score	Male	48	84.10	4037.00
-	Female	152	108.38	16,063.00
-	Total	200	-	-
Visual Analogue Scale (VAS)	Male	48	86.50	4152.00
-	Female	152	107.56	15,948.00
-	Total	200	-	-
Test statistics	U	W	Z	p
STOP-Bang Questionnaire (SBQ)	27888.000	3955.000	-2.412	0.16^*^
Apfel Simplified Risk Score	2870.000	4037.000	-2.218	0.27^*^
Visual Analogue Scale (VAS)	2985.000	4152.000	-2.001	0.45^*^

Table 4 shows statistically significant differences among age groups for all three measures. SBQ scores tended to increase with age, with participants aged 61 years and above having the highest mean rank (Mean Rank = 134.20; χ²(5) = 15.84, p = 0.007). In contrast, Apfel Simplified Risk Scores were higher in younger participants, with the 18-25 years group showing the highest mean rank (132.50; χ²(5) = 19.33, p = 0.002). Similarly, VAS scores were higher among younger participants (18-25 years; Mean Rank = 125.30) and lower in older participants (61+ years; Mean Rank = 90.70; χ²(5) = 17.41, p = 0.004). These results indicate that SBQ scores increase with age, whereas Apfel and VAS scores are higher in younger participants.

**Table 4:** Kruskal–Wallis test comparing age group differences on the STOP-Bang Questionnaire, Apfel Simplified Risk Score, and Visual Analogue Scale (N=200) Note. N = number of participants in each viral infection category; % = percentage of the total sample; Percentages are based on total N = 200; SBQ= STOP-Bang Questionnaire; APFEL= Apfel Simplified Risk Score; VAS= Visual Analogue Scale; Values are mean ranks from Kruskal–Wallis H tests; Overall test statistics are reported; Total SBQ (χ²(5) = 15.84, p= 0.001**), Total APFEL (χ²(5) = 19.33, p= 0.002**), and Total VAS (χ²(5) = 17.41, p= 0.004**); Significance levels: p <0.01**.

Age Group	N (%)	Mean Rank (Total SBQ)	Mean Rank (Total APFEL)	Mean Rank (Total VAS)
18–25 years	10 (5)	88.40	132.50	125.30
26–35 years	5 (2)	90.20	120.40	118.60
36–45 years	7 (3)	95.10	110.70	108.20
46–55 years	54 (27)	102.60	100.90	98.40
56–60 years	73 (36)	108.80	94.30	95.10
61+ years	51 (25)	134.20	85.40	90.70
Total	200	-	-	-
Test statistics	-	H	df	p
Total SBQ	-	15.842	5	0.007^**^
Total APFEL	-	19.327	5	0.002^**^
Total VAS	-	17.411	5	0.004^**^

Table 5 shows statistically significant differences in SBQ, Apfel Simplified Risk Score, and VAS scores based on medication use. Participants using insulin or other diabetes medications had the highest mean ranks on SBQ (118.30), Apfel (120.80), and VAS (121.70), followed by participants taking blood pressure medications. Participants not taking any regular medication had the lowest mean ranks on all three measures (SBQ = 82.40; Apfel = 85.60; VAS = 88.30). Kruskal-Wallis tests confirmed significant group differences for SBQ (χ²(5) = 14.72, p = 0.012), Apfel (χ²(5) = 16.32, p = 0.006), and VAS (χ²(5) = 15.49, p = 0.009). These results indicate that medication use is associated with higher scores on the SBQ, Apfel, and VAS measures, suggesting potential differences in OSA risk and postoperative nausea severity across medication groups.

**Table 5:** Kruskal–Wallis test comparing use of medication differences on the SBQ, Apfel Simplified Risk Score, and VAS (N=200) Note. N = number of participants in each viral infection category; % = percentage of the total sample; Percentages are based on total N = 200; SBQ= STOP-Bang Questionnaire; APFEL= Apfel Simplified Risk Score; VAS= Visual Analogue Scale; Values are mean ranks from Kruskal–Wallis H tests; Overall test statistics are reported; Total SBQ (χ²(5) = 14.722, p= 0.012**), Total APFEL (χ²(5) = 16.318, p= 0.006**), and Total VAS (χ²(5) = 15.486, p= 0.009**); Significance levels: p <0.05*, p <0.01**.

Medication use	N	Mean Rank (Total SBQ)	Mean Rank (Total APFEL)	Mean Rank (Total VAS)
No regular medication	10 (5)	82.40	85.60	88.30
Opioids (e.g., morphine, oxycodone, etc.)	22 (11)	95.20	97.40	99.10
Antidepressants or anti-anxiety medication	34 (17)	102.10	106.70	108.50
Blood pressure medications	78 (39)	111.90	114.20	115.40
Insulin or other diabetes medications	54 (27)	118.30	120.80	121.70
Other	2 (1)	130.00	129.50	128.50
Total	200	-	-	-
Test statistics	-	H	df	p
Total SBQ	-	14.722	5	0.012^*^
Total APFEL	-	16.318	5	0.006^**^
Total VAS	-	15.486	5	0.009^**^

Table 6 presents the results of a multiple linear regression analysis used to predict APFEL scores based on STOP-Bang, severity of nausea as measured by VAS, age, sex, medication intake, surgical history, and anesthesia history (N = 200). In general, this model was significant, as it explained 40% of the variance in the APFEL score (R² = 0.400, Adjusted R² = 0.383). The results were statistically significant (p < 0.01) for all predictors. In detail, higher STOP-Bang scores (B = 0.152) and more intense nausea (B = 0.176) were linked to higher values of APFEL, and older age to lower values of APFEL (B = -0.128). Moreover, the female gender (β = 0.125), medication, positive surgical history (β = 0.121), and prior anesthesia history (β = 0.133) also made significant contributions to the higher APFEL. This evidence indicates that there is an independent relationship between patient and perioperative factors and a predictor of postoperative outcomes, pointing to PONV risk as a multifactorial problem.

**Table 6:** Multiple regression predicting Apfel scores from STOP-Bang, VAS nausea severity, age, gender, medication use, sleep apnea diagnosis, surgical history, and anesthesia history (N = 200) Note. Multiple linear regression was conducted to identify predictors of postoperative outcomes scores; The full regression model including SBQ, Apfel risk score, and other predictors explained 40% of variance in nausea (R² = 0.400), While SBQ alone had a small correlation with nausea (r = 0.18), its contribution to the combined Model was significant; Values include unstandardized coefficients (B), 95% confidence intervals (CI), standard error (SE), standardized beta coefficients (β), and p-values; A p-value < 0.05 was considered statistically significant, N = 200.

Predictor	B	SE	β	t	p	95% CI LL	95% CI UL
Constant	5.210	0.720	-	7.236	<0.001^**^	3.792	6.628
STOP-Bang (SBQ)	0.085	0.028	0.152	3.036	0.003^**^	0.030	0.140
VAS (Nausea severity)	0.067	0.022	0.176	3.045	0.003^**^	0.024	0.110
age	-0.092	0.031	-0.128	-2.968	0.003^**^	-0.153	-0.031
gender	0.140	0.052	0.125	2.692	0.008^**^	0.038	0.242
Medication use	0.120	0.040	0.142	3.000	0.003^**^	0.041	0.199
Surgical history	0.098	0.034	0.121	2.882	0.004^**^	0.031	0.165
Anesthesia history	0.110	0.037	0.133	2.973	0.003^**^	0.037	0.183
Model summary	R	R^2^	Adjusted R^2^	SE of the estimate	-	-	-
-	0.633	0.400	0.383	2.15	-	-	-

Table 7 showed a significant association between age and being diagnosed or assessed for obstructive sleep apnea (χ²(5) = 18.24, p = 0.003). Among the 200 participants, 49 (25%) had been diagnosed or assessed for OSA, while 151 (76%) had not. No participants aged 18-25 or 26-35 were diagnosed. The first diagnosis appeared in the 36-45 years group (1, 14%), and the proportion of diagnosed participants increased in older age groups: 14 of 54 (26%) in 46-55 years, 23 of 73 (32%) in 56-60 years, and 11 of 51 (22%) in 61 years and above. These results suggest that older age groups in this sample were more likely to be diagnosed or evaluated for OSA compared to younger participants.

**Table 7:** Chi-square test of age group differences in diagnosed or evaluated for obstructive sleep apnea (n = 200). Note. Data are presented as N (%); the Chi-square test was used to assess the relationship between age and diagnosed or evaluated for Obstructive Sleep Apnea frequency; Statistical significance was considered at p < 0.05.

Age	No; N (%)	Yes; N (%)	Total	χ² (df = 5)	p
18–25 years	10 (100)	0 (0)	10	-	-
26–35 years	5 (100)	0 (0)	5	-	-
36–45 years	6 (86)	1 (14)	7	-	-
46–55 years	40 (74)	14 (26)	54	-	-
56–60 years	50 (68)	23 (32)	73	-	-
61+ years	40 (78)	11 (22)	51	-	-
Total	151 (76)	49 (25)	200	18.24	0.003^**^

Table 8 showed a significant association between age and postoperative nausea and vomiting (PONV) (χ² (5) = 21.77, p = 0.001). Among the 200 participants, 45 (23%) experienced PONV, while 155 (77%) did not. Younger participants exhibited higher rates of PONV: 7 of 10 (70%) in the 18-25 years group, 4 of 5 (80%) in the 26-35 years group, and 4 of 7 (57%) in the 36-45 years group. In contrast, PONV prevalence decreased with age: 16 of 54 (30%) in 46-55 years, 9 of 73 (12%) in 56-60 years, and 5 of 51 (10%) in those aged 61 and above. These findings suggest that younger adults in this sample were more likely to experience PONV, whereas older adults were less likely.

**Table 8:** Chi-square test of age group differences in postoperative nausea/vomiting (PONV) (n = 200) Note. Data are presented as N (%); the Chi-square test was used to assess the relationship between age and Postoperative Nausea/Vomiting frequency; Statistical significance was considered at p < 0.05.

Age	No; N(%)	Yes; N(%)	Total	χ² (df = 5)	P
18–25 years	3 (30)	7 (70)	10	-	-
26–35 years	1 (20)	4 (80)	5	-	-
36–45 years	3 (43)	4 (57)	7	-	-
46–55 years	38 (70)	16 (30)	54	-	-
56–60 years	64 (88)	9 (12)	73	-	-
61+ years	46 (90)	5 (10)	51	-	-
Total	155 (77)	45 (23)	200	21.77	0.001^**^

## Discussion

In this prospective observational study, we found a mild positive association between STOP-Bang-defined OSA risk and both predicted PONV risk and experienced nausea severity (VAS). This association remained statistically significant after adjusting for relevant demographic and clinical factors. Higher SBQ scores were associated with increased Apfel scores and VAS-measured nausea, suggesting that preoperative OSA risk may contribute to postoperative nausea and vomiting. These findings are consistent with previous literature indicating that patients with sleep-disordered breathing are at greater risk of perioperative adverse events, including PONV [16]. In our analysis, the Apfel risk score was moderately positively correlated with the severity of nausea, as measured by the VAS, which supports its ability to predict PONV. Likewise, other studies have found a significant relationship between elevated Apfel scores and a high rate of PONV, providing evidence that underpins the practicality of the scoring system in perioperative risk stratification [17]. The link between OSA and PONV may involve intermittent hypoxia, altered autonomic function, and increased sympathetic activity, which can affect gastrointestinal motility and chemoreceptor sensitivity. Additionally, OSA-related neuroendocrine fluctuations, including cortisol and catecholamine changes, may exacerbate postoperative nausea and vomiting. These mechanisms provide a plausible explanation for the observed association between OSA risk and PONV.

In our study, the SBQ was significantly higher in females than in males because they had a higher screening-based risk of OSA. This result is consistent with pre-existing literature, as OSA in women remains underrecognized and may present differently compared to its occurrence in men, which is why gender-specific differences in the diagnosis and treatment of OSA are crucial [18]. In our study, women were substantially more likely to exceed the Apfel thresholds. They reported more severe postoperative nausea and vomiting on the VAS, indicating both increased predicted and demonstrated PONV risk. This concurs with past evidence noting that female sex is the most powerful independent risk factor of PONV, hence the requirement of gender-specific preventive measures [19].

In our research, the difference in SBQ scores between older and younger participants was significant, demonstrating that the risk of OSA increases with age. Similar results have also been reported in other studies, which indicate a steadily increasing prevalence of OSA with advancing age [20]. SBQ scores increased with age, while younger participants had higher Apfel scores and VAS severity, suggesting that age is inversely related to PONV risk despite being positively associated with OSA risk, consistent with previous studies [21].

In our study, participants taking blood pressure and diabetes medications had the highest SBQ score compared to those who did not take regular medication, which had the lowest risk. This is in line with the results that OSA severity is associated with resistant hypertension despite intensive treatment, emphasizing the OSA severity and blood pressure control variability [22]. In our analysis, the Apfel risk scores and the severity of nausea and vomiting were more significant in patients taking antihypertensive and hypoglycemic medications. This finding aligns with reports that significant decreases in blood pressure during anesthesia are also associated with an increased incidence of PONV, supporting the importance of cardiovascular susceptibility [23].

In line with our results, our predecessors also highlighted the idea that patients with obstructive sleep apnea have a greater risk of developing perioperative complications, including PONV. Our finding that STOP-Bang scores were significantly associated with increased APFEL risk scores supports the importance of sleep-disordered breathing as a major predictor of PONV [16]. We found that higher VAS nausea severity significantly predicted higher Apfel scores. This is consistent with previous findings that nausea severity is powerfully correlated with an elevated risk of PONV, which justifies its use as a predictor in this clinical context [17]. In line with past studies, younger patients in our sample had higher APFEL scores, linked to a high risk of postoperative nausea and vomiting, whereas age seemed to play a minor protective role. This finding aligns with previous studies indicating an inverse relationship between age and PONV prevalence [21]. Our regression analysis also showed that gender is a significant predictor of the risk of PONV, with females scoring higher. This is supported by prior large-cohort studies and recommendations, all of which support female sex as a potential robust and independent risk of PONV [19]. Our results indicated that those patients who used regular medications were at considerable risk of developing PONV. This can be proven by the fact that there has been previous evidence showing that significant reductions of blood pressure during surgery, which tend to be above average levels in some patients taking antihypertensive medication, are highly linked to the incidence of PONV [23]. Our results showed that a history of previous surgery was a strong predictor of PONV. This aligns with previous research, which has indicated that, despite surgical site and a history of PONV having limited predictive capabilities, prior exposure was included as a factor when calculating multifactorial PONV scores [24]. Our results showed that prior anesthesia exposure was predictive of PONV. This is aligned with evidence-based guideline reviews, which identify prior PONV and anesthesia-related exposures (e.g., opioids, inhalational agents, nitrous oxide, and prolonged anesthesia) as significant risk factors, underscoring the necessity of considering the anesthetic history when planning prophylaxis [25].

In our research, the prevalence of obstructive sleep apnea was higher among older participants, with the proportion diagnosed increasing with age. This finding is consistent with prior evidence indicating that OSA prevalence increases with age, and therefore, age is a significant risk factor for OSA [20]. We have found that younger patients had higher incidences of PONV, and this is in agreement with a similar study done on patients with orthognathic surgery, as age was found to be a significant risk factor. Those younger were more prone to postoperative nausea and vomiting [21].

Overall, our findings support the clinical relevance of STOP-Bang screening to identify patients at increased risk for PONV and guide targeted preventive strategies.

Limitations

This research has several limitations. First, the sample was selected through non-random convenience sampling in core hospitals in Lahore, which may limit the generalizability of the findings. Second, the use of self-reported history and questionnaire-based instruments without follow-up polysomnography could have introduced reporting bias, misclassification of OSA risk, and recall bias for postoperative nausea and vomiting. Third, potential confounding factors such as body mass index (BMI), smoking status, and the specific type of laparoscopic procedure were not controlled, which may have influenced the observed associations. Fourth, intraoperative variables that could affect PONV, including anaesthesia duration, anaesthesia type, and fluid management, were not assessed. Fifth, the study investigated only associations and therefore cannot establish causal relationships due to its observational design. Sixth, the use of the Apfel score may have underestimated PONV risk because patients with prior PONV were excluded. Seventh, the sample size (n = 200) was smaller than the ideal of 384, which may have weakened the observed correlations. Finally, the predominance of female participants may have contributed to gender-related variations in STOP-Bang, Apfel, and VAS scores.

Future directions

Future research should incorporate objective diagnostic methods, such as polysomnography, to validate questionnaire-based OSA screening in surgical populations. The external validity of the findings could be enhanced by increasing the sample size and using populations more representative of various regions in Pakistan. Assessing intraoperative and postoperative strategies, such as the anesthetic agent used or prophylactic antiemetics, as well as postoperative care measures, would also give a more complete picture of how OSA risk interacts with PONV. Randomized controlled trials examining whether focused perioperative efforts on high OSA-risk patients translate into a reduction in PONV would also be of clinical interest.

## Conclusions

This study demonstrates a mild to moderate positive association between STOP-Bang-detected OSA risk and the occurrence of postoperative nausea and vomiting (PONV) in patients undergoing laparoscopic surgery. STOP-Bang scores were positively associated with Apfel risk and nausea severity, independent of other patient-related and clinical factors. PONV incidence was higher among younger patients and females, whereas OSA risk was higher among older patients. These findings support the clinical utility of integrating OSA risk screening into routine preoperative assessments, thereby enhancing risk stratification and informing individualized preventive strategies. Preoperative STOP-Bang screening should be routinely implemented in laparoscopic surgical candidates to identify patients at high risk of PONV and guide prophylactic interventions.
